# Processing-induced hydrolysis and degradation pathways of glucotropaeolin in edible garden nasturtium (*Tropaeolum majus L.*) extracts

**DOI:** 10.1016/j.fochx.2026.104162

**Published:** 2026-07-01

**Authors:** Lucie Chocholoušová Havlíková, Marija Dmytryšyn, Hana Kočová Vlčková, Štefan Kosturko, Petr Chocholouš, František Švec

**Affiliations:** Charles University, Faculty of Pharmacy in Hradec Králové, Department of Analytical Chemistry, Heyrovského 1203, 500 03 Hradec Králové, Czech Republic

**Keywords:** Garden nasturtium, Benzyl isothiocyanate, Kinetics, Myrosinase, Stability

## Abstract

Garden nasturtium (*Tropaeolum majus L.*) is a rich source of bioactive compounds, particularly glucotropaeolin, and is increasingly used in functional foods. Maintaining glucosinolate stability during processing is critical for ensuring consistent bioactivity. This study systematically investigated the kinetics and mechanism of glucotropaeolin degradation in edible extracts obtained from seeds, leaves, and flower buds under typical processing and storage conditions. Glucotropaeolin hydrolysis was primarily driven by myrosinase, producing benzyl isothiocyanate, which then degraded to form benzyl thiocarbamate and 1,3-dibenzyl-2-thiourea. Untreated or lyophilised extracts showed rapid glucotropaeolin degradation, whereas boiling-induced myrosinase inactivation substantially improved glucotropaeolin stability. Additionally, an analysis of commercial garden nasturtium extracts revealed surprisingly low levels of glucotropaeolin and benzyl isothiocyanate, highlighting the impact of industrial processing. These findings provide insight into the mechanism of glucotropaeolin stability and highlight processing strategies for preserving bioactive glucosinolates, supporting the development of functional foods with predictable bioactive profiles.

## Highlights

• Regulation of extraction conditions as a strategy for enhancing stability of glucosinolates and isothiocyanates in functional extracts.
• Kinetics of glucotropaeolin extraction and its hydrolysis.
• UHPLC-MS/MS reveals benzyl isothiocyanate degradation intermediates.
• Practical framework for preparing nasturtium extracts with controlled bioactive profiles.

## Graphical abstract

**Figure:**
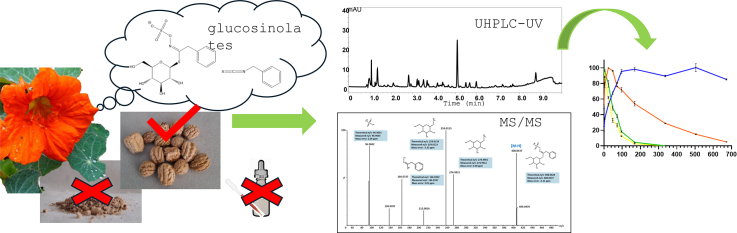

## 1 Introduction

The garden nasturtium (*Tropaeolum majus L.*, Tropaeolaceae Juss. ex DC.) is an edible plant. Its leaves have a slightly sweet, peppery flavour, while its bright orange or red flowers taste milder. It is often used in salads and soups. In traditional medicine, garden nasturtium is said to have antioxidant, anti-inflammatory, diuretic, and antibacterial properties (Bazylko et al., 2013; Brondani et al., 2016; Frei et al., 2024). The plant contains a variety of bioactive compounds, such as phenolic compounds, vitamin C, minerals, fatty acids, carotenoids, terpenoids, phyloxantobillins, and glucosinolates (GSs) (Frei et al., 2024). These are negatively charged, sulphur-containing, hydrophilic secondary metabolites that are found in the Brassicaceae family and related species. In garden nasturtium, they are represented by glucotropaeolin (GT) (Česlová et al., 2023).

The hydrolysis of GT in plants is catalysed by the enzyme myrosinase (Ghawi et al., 2012). Previous studies revealed significant variations in myrosinase activity both among species within the Brassicales order (Piekarska et al., 2013) and among cultivars within the same species (Piekarska et al., 2013; Restović et al., 2024; Van Eylen et al., 2007; Vieites-Outes et al., 2016). Additionally, a gradual decrease in myrosinase expression was observed during plant growth and development (Restović et al., 2024; Wielanek et al., 2004). In intact plant tissues, GSs and myrosinase exist in distinct cellular structures. Following tissue disruption (e.g., slicing, grinding, or chewing), the enzyme comes into contact with its substrates, initiating GSs hydrolysis and the formation of a range of breakdown products (Hanschen et al., 2014). The resulting products of the enzymatic hydrolysis are unstable aglycones that subsequently undergo a Lossen rearrangement to produce isothiocyanates (Blažević et al., 2020; Hanschen et al., 2018). Hanschen et al. investigated the low stability and high reactivity of isothiocyanates (ITCs) and the release of their degradation products in model systems and selected plants of the Brassicaceae family under various conditions, including temperature and pH (Andernach et al., 2024; Hanschen et al., 2012; Hanschen et al., 2018; Krell et al., 2022; Kühn et al., 2018; Zhang et al., 2026). ITCs were observed to readily react with strong nucleophiles, such as free thiol or amino groups. The reaction rate was influenced by pH, temperature, and the structural characteristics of the side chains. Thiol reactions generally occur more quickly in biological systems and food matrices (Andernach et al., 2024; Hanschen et al., 2012; Hanschen et al., 2014). ITCs degrade to form amines, thiocarbamates, thiourea derivatives, and a number of volatile compounds (Andernach et al., 2024).

In humans, benzyl isothiocyanate (BITC) is primarily metabolized via the mercapturic acid pathway. This involves initial conjugation with glutathione, followed by the subsequent formation of dithiocarbamate metabolites (Wang et al., 2025; Ye et al., 2002). However, unconjugated BITC was alsodetected in urine, and a small amount of volatile isothiocyanates may be exhaled (Kühn et al., 2018).

Similar to GCs, ITCs exhibit multiple bioactive effects, including antimicrobial, anti-inflammatory, chemoprotective (Hanschen et al., 2014; Vrca et al., 2022), and anthelmintic activity (Jang et al., 2010; O'Hare et al., 2008; Sofrata et al., 2011). BITC has been shown to be effective against *Staphylococcus aureus* and *Escherichia coli,* and to induce chemoprotective enzymes with anti-cancer effects (Dinh et al., 2021; Veeranki et al., 2015; Vrca et al., 2022).

The antioxidant and antibacterial effects of phenolic compounds present in garden nasturtium extracts against Gram-negative bacteria were experimentally confirmed (Bortolini et al., 2022). The pronounced antioxidant and anti-inflammatory effects are linked to COX-1 and COX-2 inhibition, which is mediated by quinic and cinnamic acid esters (e.g., chlorogenic acid), vitamin C, isoquercitrin, and phytoxantobillins (Bazylko et al., 2013; Frei et al., 2024; Setyaningsih et al., 2025). This is therapeutically relevant for urinary tract infections (Frei et al., 2024).

Additional important sources of GT include garden cress, papaya, and maca (Lucarini et al., 2026; O'Hare et al., 2008; Xu et al., 2021). In these species, GT concentrations are generally higher in seeds than in leaves (O'Hare et al., 2008). Substantial amounts of GT were reported in papaya latex (Burow et al., 2007), whereas in maca, GT is the predominant glucosinolate, particularly in the hypocotyls (Yábar et al., 2011). GT-rich extracts from garden cress demonstrated significant anti-inflammatory effects in the treatment of tendinopathy (Lucarini et al., 2026). Similarly, maca was reported to exhibit potent antioxidant and anti-inflammatory activities (Ulloa Del Carpio et al., 2024).

The content of secondary metabolites in garden nasturtium tissues is significantly influenced by climate and growing conditions (Česlová et al., 2023; Frei et al., 2024). Drops (often in the tincture form) and capsules containing declared content of nasturtium extract are available on the market as food supplements. They cannot make health claims and only provide general information on natural antimicrobial activity and phenolic content.

The present study systematically investigated the stability and kinetics of GT hydrolysis in the garden nasturtium seeds, leaves, and flower buds that have been subjected to various pretreatment strategies. The aim was to optimize extracts with a high GT and BITC content for use in functional foods. Due to growing demand from consumers for plant-based health products and the popularity of extracts prepared at home, the stability of GT in real hydroethanolic extracts stored at room temperature was examined in particular. This enabled the evaluation of release and degradation pathways, and the quantification of GT, BITC, and related breakdown products, including the kinetic analysis of intermediate reactions. Additionally, the quality and authenticity of commercial nasturtium-containing products were assessed by determining their glucosinolate content.

As outlined in the introduction, numerous studies investigated the hydrolysis of GSs and the reactivity of ITCs in model systems or plant tissues. However, the knowledge of the content and subsequent reactions of ITCs in *Tropaeolum majus* L. extracts is still limited. This work contributes to the evidence-based development of stable, high-quality plant-derived ingredients, supporting the sustainable utilization of GSs and ITCs in future food systems.

## 2 Materials and methods

### 2.1 Chemicals and materials

Glucotropaeolin potassium salt (99.8% HPLC; GT) was purchased from PhytoLab (Germany). Benzyl isothiocyanate (97.5%, GC; BITC), benzyl thiocyanate (95.0%, GC; BTC), benzyl cyanide (97.5%, GC; BC), 1,3-dibenzyl-2-thiourea (*N*,*N*′-dibenzylthiourea, 97.0%, GC; DP2), benzylamine (95.0%, GC), and benzyl alcohol (96.3%, GC) were purchased from Sigma-Aldrich (Czech Republic). Formic acid, ortho-phosphoric acid, LC–MS grade acetonitrile, pure ethanol, and LC–MS grade methanol were provided by Merck (Czech Republic). Ultra-pure water was obtained from a Millipore Milli-Q reverse osmosis system (Bedford, MA, USA) immediately prior to use. Garden nasturtium seeds were purchased from Milota in the Czech Republic. Seven garden nasturtium drop samples were purchased from the local market (more details are presented in Table 1).

**Table 1:**
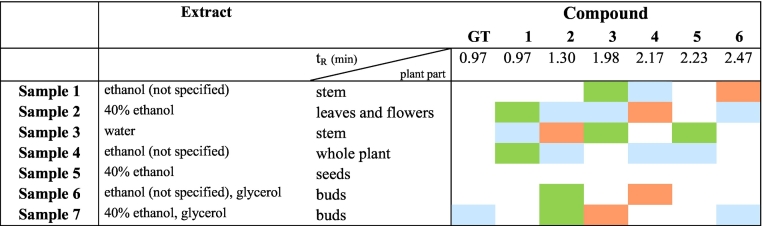
A description of the samples and an overview of the content of the analytes of interest in the samples determined semi-quantitatively by UHPLC-HRMS. High content (red), significant content (blue), detectable content (green), and no content (white).

### 2.2 Sample preparation

Various extraction procedures were implemented to evaluate GT kinetics and quantify GT content in seed extracts, plant tissues, and food supplements. Extracts of garden nasturtium seeds were prepared from: (i) whole seeds (Extract A), (ii) from coarsely ground seeds to an average particle size of 0.3 mm, followed by ultrasound-assisted extraction for 10 min, including 4 types of pretreatment before grinding (Extracts BB, BS, BL, and B), and (iii) from fresh leaves and flower buds. To enhance clarity of the multistep procedures, a summary of the individual methods of extract preparation is provided in Table S1.

For garden nasturtium Extract A, 15.0 g garden nasturtium seeds were weighed and immersed in a closed container with 100.0 mL of a 50% aqueous ethanol (hereinafter referred to as only 50% ethanol). The contents were stirred gently twice a day. At given time intervals over a period of 16 weeks, an aliquot was taken and filtered through a 0.22 μm nylon filter. It was then diluted tenfold with 70% ethanol and analysed using ultra-high performance liquid chromatography (UHPLC-UV). For the ultra-high performance liquid chromatography – high-resolution mass spectrometry (UHPLC-HRMS) analysis, Extract A was diluted one hundred times with 70% ethanol.

To obtain the B series extracts, the garden nasturtium seeds were pretreated in three different ways or used directly. To produce Extract BB, 50 g of seeds were immersed in 100.0 mL boiling deionised water and kept at this temperature for 5 min. The liquid was then decanted, and the seeds were dried at room temperature. Extract BS was obtained via steam boiling. Seeds (50.0 g) were placed on a perforated tray positioned above 100.0 mL boiling water, ensuring that the steam reaching the tray was at a temperature of 70 °C. The steaming process was stopped after 15 min, after which the seeds were dried at room temperature. Extract BL was prepared by freeze-drying. This approach involved lyophilising 50.0 g of seeds for 24 h. The seeds were frozen at −29 °C and lyophilized under deep vacuum at −55 °C using a Freeze Dryer Heto PowerDry LL 3000 (Thermo, Czech Republic) until a constant weight was achieved, which required about 24 h. No pretreatment of the seeds was used to obtain Extract B.

Then, 50.0 mL of 70% ethanol was added to 0.5 g of either pre-treated or untreated garden nasturtium seeds, which had been coarsely ground to an average particle size of 0.3 mm using an IKA A11 basic mill (Fischer Scientific, Czech Republic). This was followed by ultrasound-assisted extraction for 10 min. The tube was then placed in a dark location and shaken periodically. Aliquots (0.5 mL) of all extracts were taken at 0, 4, 24, 48, 72, and 96 h, as well as after 1, 2, 3, and 4 weeks. These aliquots were filtered through a 0.22 μm nylon filter and subjected to the UHPLC-UV analysis. To investigate the natural variation in GT levels, extract B was prepared in triplicate from a single batch and compared with three independent seed batches to evaluate differences in GT content between batches.

Extracts were also obtained from the fresh leaves and flower buds of garden nasturtium plants grown from the same batch of seeds as those used in the other experiments. Ten leaves or similarly ten buds were pooled and homogenized. The plant material was chopped into pieces approximately 0.5 cm in size. An aliquot of 0.1 g of the leaves or buds was weighed, extracted with 10.0 mL 70% ethanol, and subjected to ultrasound-assisted extraction for 10 min. The sample was shaken gently twice a day. At given time intervals, the samples were collected, filtered through a 0.22 μm nylon filter and subjected to UHPLC-UV analysis.

Finally, the garden nasturtium drops and extracts (Samples 1–7) were purchased from local markets in the Czech Republic. The food supplements were filtered through a 0.22 μm nylon filter and analysed using UHPLC-UV. For UHPLC-HRMS analysis, the supplements were diluted tenfold with 70% ethanol.

### 2.3 Ultra-high performance liquid chromatography UV detection (UHPLC-UV) and ultra-high performance liquid chromatography – High-resolution mass spectrometry (UHPLC-HRMS)

The conditions for UHPLC-UV and UHPLC-HRMS are outlined in the Supplementary material (S1.2., S1.3., S2.1., and Fig. S1). The applicability of the UHPLC-UV method was verified through system suitability testing (SST) and method validation (Supplementary material S1.4., S2.2., Tables S2 and S3).

### 2.4 Standard solutions

Standard solutions of 1 mg/mL of BITC, BTC, and BC were prepared individually in methanol and GT in 70% ethanol, respectively. These solutions were stored at −20 °C for up to two weeks for BITC, and for up to one month for the other compounds. Working standard solutions were prepared daily by diluting the standard solutions to the desired final concentrations with 70% ethanol. Working solutions for the system suitability test (Supplementary material S2.2.) were prepared at the following concentrations: 50 μg/mL for GT, 25 μg/mL for BC, 12.5 μg/mL for BTC, and 25 μg/mL for BITC. Working solutions for the degradation products analysis were prepared at the following concentrations: 50 μg/mL for benzylamine, 10 μg/mL for 1,3-dibenzyl-2-thiourea, and 25 μg/mL for benzyl alcohol. The GT working solution for the UHPLC-HRMS analyses was prepared at a concentration of 5 μg/mL. The stability of GT and BITC in 70% ethanol during storage at room temperature is summarized in the Supplementary Material (S2.3., Table S4).

### 2.5 Comparison of myrosinase activity

Myrosinase activity was determined using a modified method based on Kleinwächter (Kleinwächter et al., 2008). 0.5 g of coarsely ground untreated seeds were sonicated for 10 min in 10 mL of 25 mM phosphate buffer (pH 6.0) at 4 °C, followed by centrifugation at 2000 ×*g* for 5 min at 4 °C. The supernatant was filtered through a 0.45 μm nylon filter. Then, 2.5 mL of the filtrate was applied to a PD-10 desalting column (GE Healthcare Biosciences) to remove low-molecular-weight compounds. The enzyme fraction was eluted with 3.5 mL of phosphate buffer and used directly for myrosinase assay. The enzyme volume for each reaction was optimized to 50 μL. The reaction was monitored by HPLC-UV through the decrease in the GT content, which served as substrate. Enzyme activity was expressed in international units (U). One unit of myrosinase activity was defined as the amount of enzyme that catalysed the hydrolysis of 1 μmol of GT per min (1 U = 1 μmol/min) (Hinojosa-Luna et al., 2026). The reactions were performed with a total reaction volume of 600 μL and an initial GT concentration of 0.2 mM.

The thermal stability of myrosinase was evaluated by incubating the enzyme preparation at different temperatures (30, 40, 50, 60, 70, and 80 °C) for 20 min. To further assess the effect of sample processing, myrosinase was also isolated from pretreated seeds.

Additionally, the impact of the extraction medium on myrosinase activity was examined using phosphate buffer (pH 6.0), distilled water, absolute ethanol, and aqueous or buffer ethanol solutions with 50% or 70% (*v*/v) ethanol prepared either in water or in phosphate buffer.

### 2.6 Statistical analysis

**Fig. 1:**
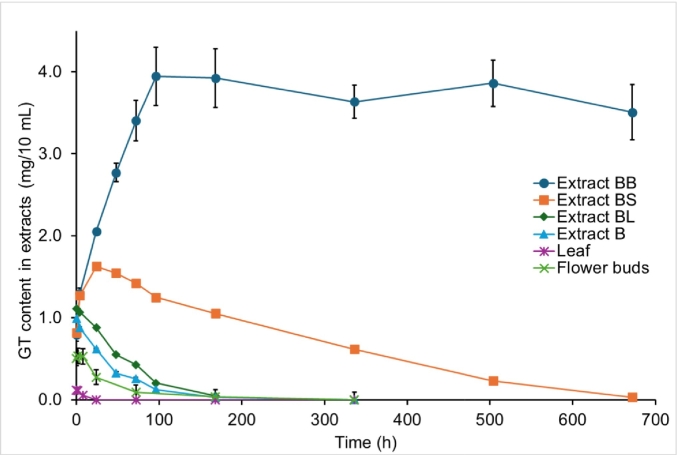
The time course of glucotropaeolin release and hydrolysis in extracts (n = 3).

**Fig. 2:**
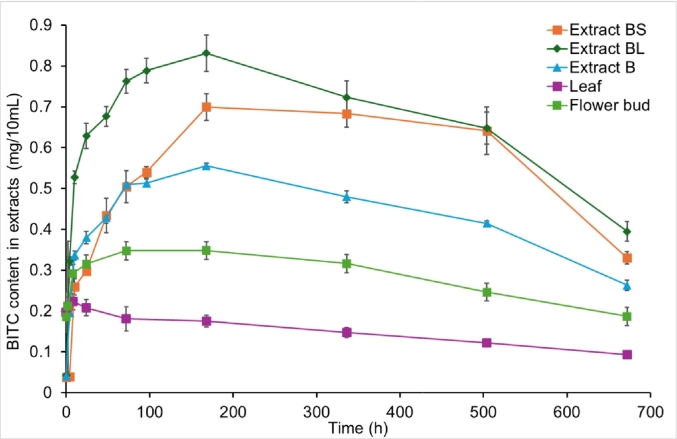
The time course of the release and hydrolysis of benzyl isothiocyanate in extracts (*n* = 3).

**Fig. 3:**
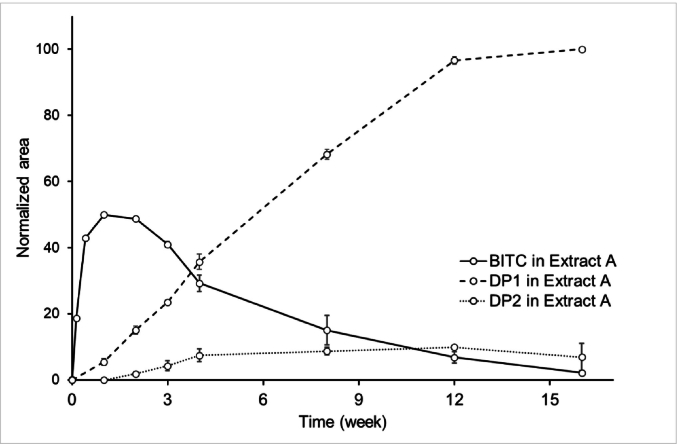
The time course of hydrolysis of benzyl isothiocyanate and the formation of DP1 and DP2 in Extract A. The maximal peak area of DP1 (dotted line) is set as 100, all other values are normalized (*n* = 3).

All experiments were performed in triplicate unless otherwise stated. Results are expressed as the mean ± SD (standard deviation). The compounds were identified by comparing their retention times and UV spectra with those of the corresponding analytical standards. Quantification of these compounds was achieved using calibration curves with UHPLC-UV (Table S3). The identity of the BITC degradation intermediates has been revealed by UHPLC-HRMS. Furthermore, the results were normalized to a scale of 100 to express the kinetic data (Fig. 1, Fig. 2, Fig. 3, Fig. 7).

## 3 Results

### 3.1 Sample preparation

The four pretreatment methods described in section 2.2 were evaluated to determine their impact on GT content. Analysis of the decanted boiling water adjusted to 100.0 mL for Extract BB revealed a GT leaching loss of 0.6 ± 0.05 mg/g dry weight. The stability of GT in boiling water for 10 min was verified experimentally. During this process, the GT concentration decreased by less than 5%, indicating no significant degradation under the applied conditions. In contrast to boiling, the condensed water collected after steaming during the preparation of Extract BS contained GT concentrations below the HPLC-UV limit of detection (LOD), suggesting minimal GT leaching from the seeds during this process. Aliquots were collected at regular time intervals over a period of 4 weeks (Table S1), filtered, and analysed by UHPLC-UV. These results were then used to evaluate the kinetics of GT hydrolysis and to monitor degradation intermediates.

### 3.2 Glucotropaeolin content in extracts

We simulated the release and hydrolysis rates of GT during maceration, the preferred method of preparing garden nasturtium extracts. The first series of extracts (Extract A) was prepared simply by macerating the untreated seeds in 50% ethanol, following the instructions provided with the commercial product. The maximum level of GT in Extract A was observed 72 h after preparation, corresponding to 0.05 ± 0.2 mg/g seeds.

The second series of extracts was prepared using the procedure outlined in section 2.2. for garden nasturtium Extract B, preceded by three different pretreatment methods aimed at accelerating the extraction process. The maximum GT level in the boiled samples (Extract BB) was reached after seven days of maceration and remained at approximately 85% of the maximum value thereafter. In the steam-treated samples (Extract BS), the maximum GT level was reached after 24 h, followed by a rapid decrease (Fig. 1). In the untreated (Extract B) and the lyophilised seeds extracts (Extract BL), the GT content was highest initially and subsequently decreased significantly. After one week of maceration, the GT content in all these samples fell below the detection limit (Fig. 1). The highest absolute GT content was found in Extract BB, corresponding to 39.9 ± 3.6 mg/g of GT in the dry seeds, followed by Extract BS, corresponding to 16.3 ± 0.2 mg/g of GT. The GT content in Extracts B and BL, expressed relative to the sample weight, corresponded to 25% and 28% of the amount quantified in Extract BB, respectively, indicating rapid GT hydrolysis. The estimated pseudo-first-order reaction rate constants (*k*) were 0.0034, 0.016, and 0.021 h^−1^ for extracts BS, BL, and B, respectively. These values correspond to the half-lives (t_1/2_) of 203, 43, and 33 h, respectively. The correlation coefficients of the linear regression of ln(C_0_/C_t_) versus time were all greater than 0.986 for all samples.

For comparison, the GT content of the leaves was 1.1 ± 0.2 mg/g at the initial stage. Similarly, the GT content in the flower buds was 5.3 ± 0.5 mg/g. Fig. 1 shows the time course of the change in GT content in extracts from garden nasturtium seeds, leaves, and flower buds. The highest GT contents achieved in the extracts are presented in Table 2.

**Table 2:** The content of glucotropaeolin (GT) and benzyl isothiocyanate (BITC) in extracts at the time of the maximum level (calculated to one gram of dried material). The amount is corresponding to the maximal level of GT during the extract determination.

Extract		Compound			
		Time (h)	GT^⁎^ (mg/g d.w.)	Time (h)	BITC (mg/g d.w.)
Extract BB		96	39.9 ± 3.6	168	0.5 ± 0.04
Extract BS		24	16.3 ± 0.2	168	7.0 ± 0.3
Extract BL		0	11.1 ± 0.4	168	8.3 ± 0.7
Extract B	Batch 1	0	10.0 ± 0.2	168	5.6 ± 0.4
Extract B	Batch 2	0	15.2 ± 2.2	168	7.2 ± 0.6
Extract B	Batch 3	0	7.1 ± 1.1	168	3.6 ± 0.3
Leaf		0	1.1 ± 0.2	8	2.2 ± 0.2
Flower bud		2	5.3 ± 0.5	168	3.5 ± 0.3
Extract A		72	0.05 ± 0.2	168	5.3 ± 0.4

*relative content.

It should be noted that the reported GT levels cannot be considered the absolute GT content of dry seeds due to the rapid onset of GT hydrolysis. In the case of Extract BB, loss during boiling must also be considered.

### 3.3 Hydrolysis of glucotropaeolin in garden nasturtium extracts

**Fig. 4:**
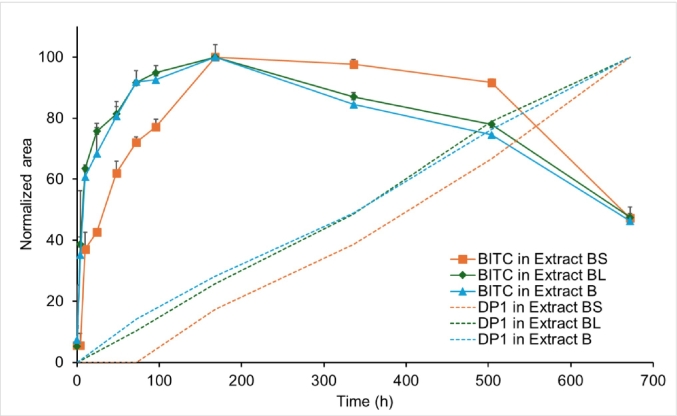
The time course of hydrolysis of benzyl isothiocyanate and its degradation to DP1 in leaves, flower buds, and Extracts BS, BL, and B. The maximum peak area in each sample is set to be 100, all other values are normalized (n = 3).

**Fig. 5:**
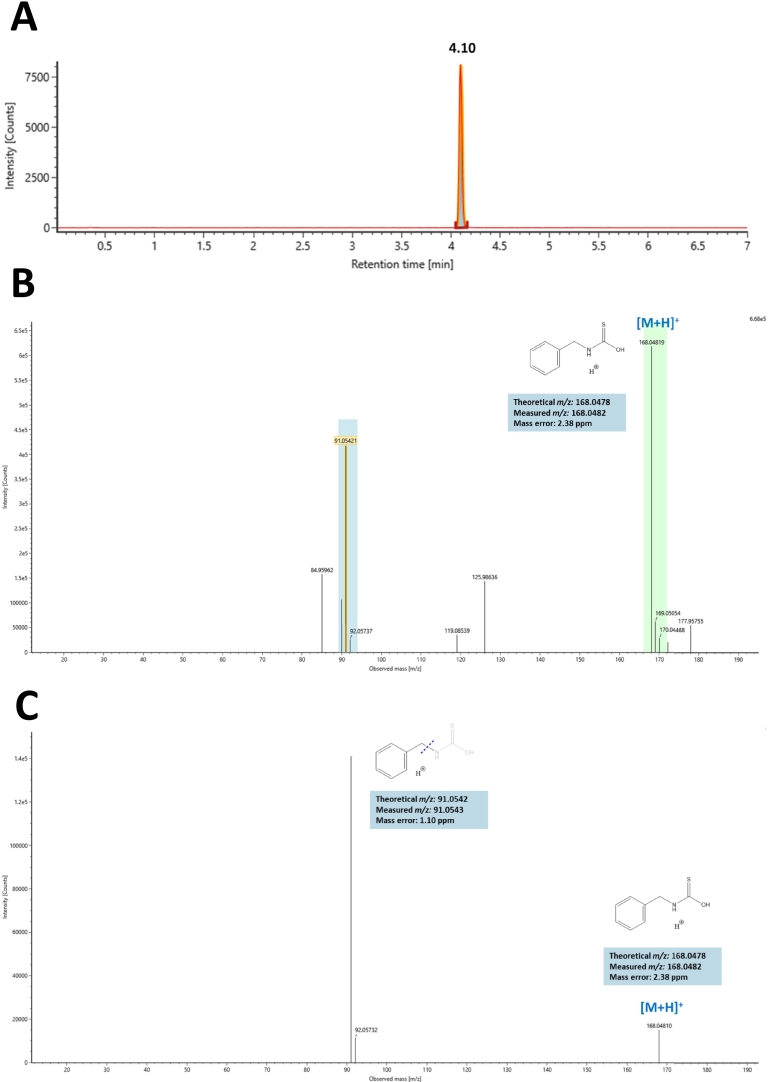
UHPLC chromatogram and MS spectra with low and high collision energy of benzyl thiocarbamate intermediate (DP1, retention time 4.10 min) in Extract A. Reconstructed ion chromatogram (RIC) of *m/z* 168.0478 (A), low energy MS spectrum (B), and high energy MS spectrum (C).

**Fig. 6:**
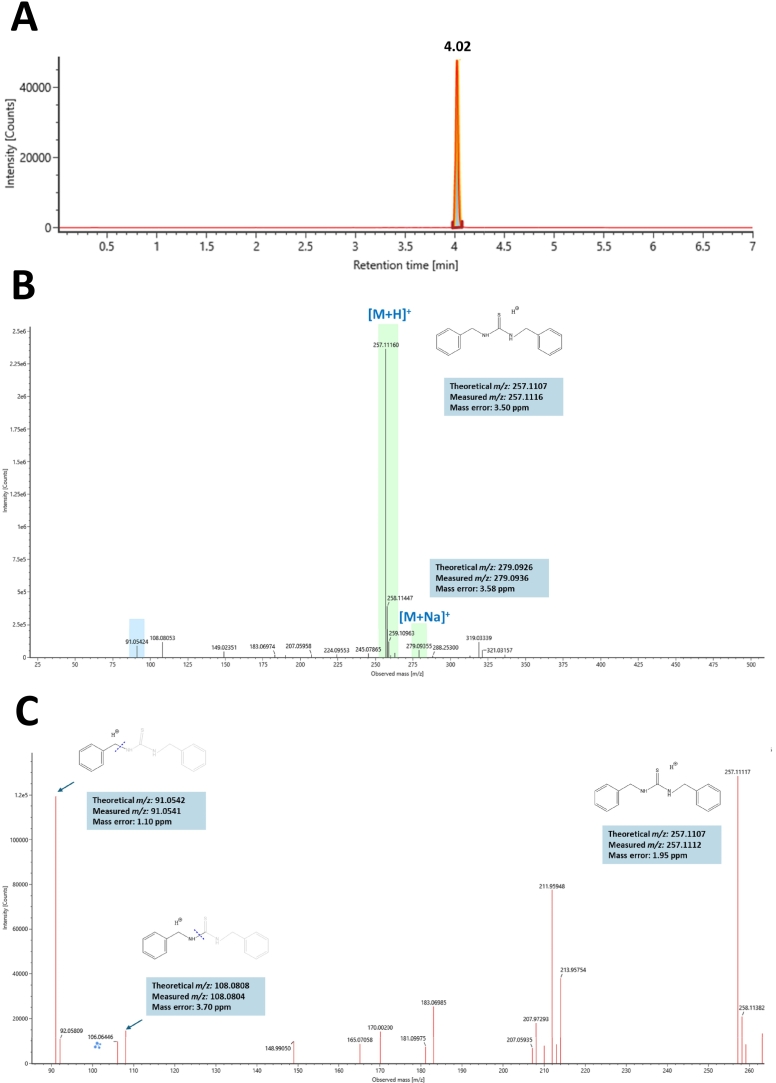
UHPLC chromatogram and MS spectra with low and high collision energy of 1,3-dibenzyl-2-thiourea (DP2, retention time 4.02 min) in Extract A. Reconstructed ion chromatogram (RIC) of *m/z* 257.1107 (A), low energy MS spectrum (B), and high energy MS spectrum (C).

**Fig. 7:**
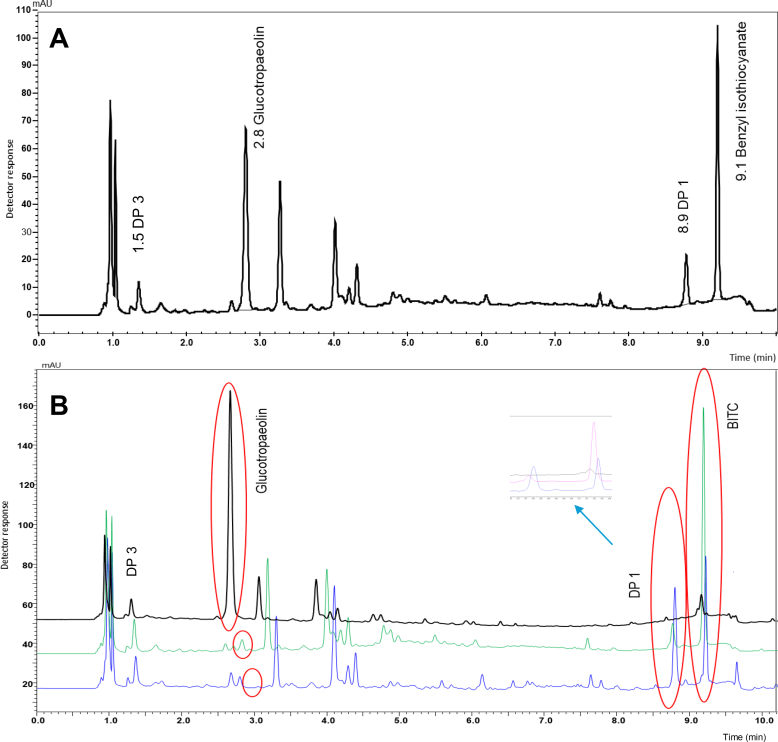
UHPLC-UV analysis of Garden nasturtium seed extracts. Extract BS at extraction time 168 h (A) and Extract B at extraction time 0 h (black line), 168 h (green line), and 672 h (blue line). In box - zoom-in of the decrease of BITC and increase of DP1 (B). Conditions: Supelco Ascentis Express Phenyl-Hexyl column 100 × 4.6 mm, 5 μm, using a gradient of 0.085% aqueous phosphoric acid and acetonitrile, specified in section 2.3., column oven at 30 °C, and UV detection at 220 nm. (For interpretation of the references to colour in this figure legend, the reader is referred to the web version of this article.)

GT was exclusively hydrolysed to BITC in all the extracts prepared from garden nasturtium seeds. The highest level of BITC was observed in all extracts prepared from the seeds after an extraction time of 168 h, after which the level decreased (Fig. 2). After this time, the BITC content was found to be 7.0 ± 0.3 mg/g in Extract BS, 8.3 ± 0.7 mg/g in Extract BL, 5.6 ± 0.4 mg/g in Extract B, and 0.5 ± 0.04 mg/g in Extract BB. GT in leaves and flower buds was likewise hydrolysed exclusively to BITC. The maximum BITC levels are summarized in Table 2. To gain a deeper insight into the decomposition pathways of BITC, its stability profile was investigated using Extract A stored at room temperature for 16 weeks. The maximum level of BITC was observed after time 168 h, corresponding to 5.27 ± 0.4 mg/g. Degradation followed pseudo-first-order kinetics with a rate constant of k = 0.2 week^−1^ (t₁/₂ = 4 weeks; R^2^ = 0.982). Chromatographic analysis revealed two degradation intermediates: DP1 (retention time 8.70 min; UV λ_max_ 244 nm), detected after 48 h, and DP2 (retention time 8.56 min; UV λ_max_ 190 nm), which was detected at time 168 h. DP2 was identified as 1,3-dibenzyl-2-thiourea based on comparison with a reference standard. A minor product, benzylamine (DP3, retention time 1.5 min; UV λ_max_ 190 nm), was also detected but did not increase after 168 h. Fig. 3 illustrates the time course of the formation of the degradation intermediates in Extract A. As no analytical standard for DP1 was available, the quantitative analysis of BITC degradation was performed using normalized peak areas. The maximum detector response (peak area) for the chosen compound was set to 100, and all other peak areas were expressed relative to this value (Fig. 3, Fig. 4). UHPLC-HRMS analysis of Extract A shown in Fig. 5 tentatively annotated DP1 as a benzyl thiocarbamate intermediate (*m/z* 168.0478). DP2 was annotated as 1,3-dibenzyl-2-thiourea (*m/z* 257.1107) (Fig. 6). The formation of DP1 was also observed in Extracts BS, BL, and B (Fig. 4). Fig. 7a shows the chromatogram of Extract BS at an extraction time of 168 h, which exhibits a significant GT and BITC content. Fig. 7b presents chromatograms illustrating the gradual hydrolysis of GT into BITC and the formation of DP1 in Extract BS throughout the experiment.

### 3.4 Profile of compounds in commercially available food supplements

**Fig. 8:**
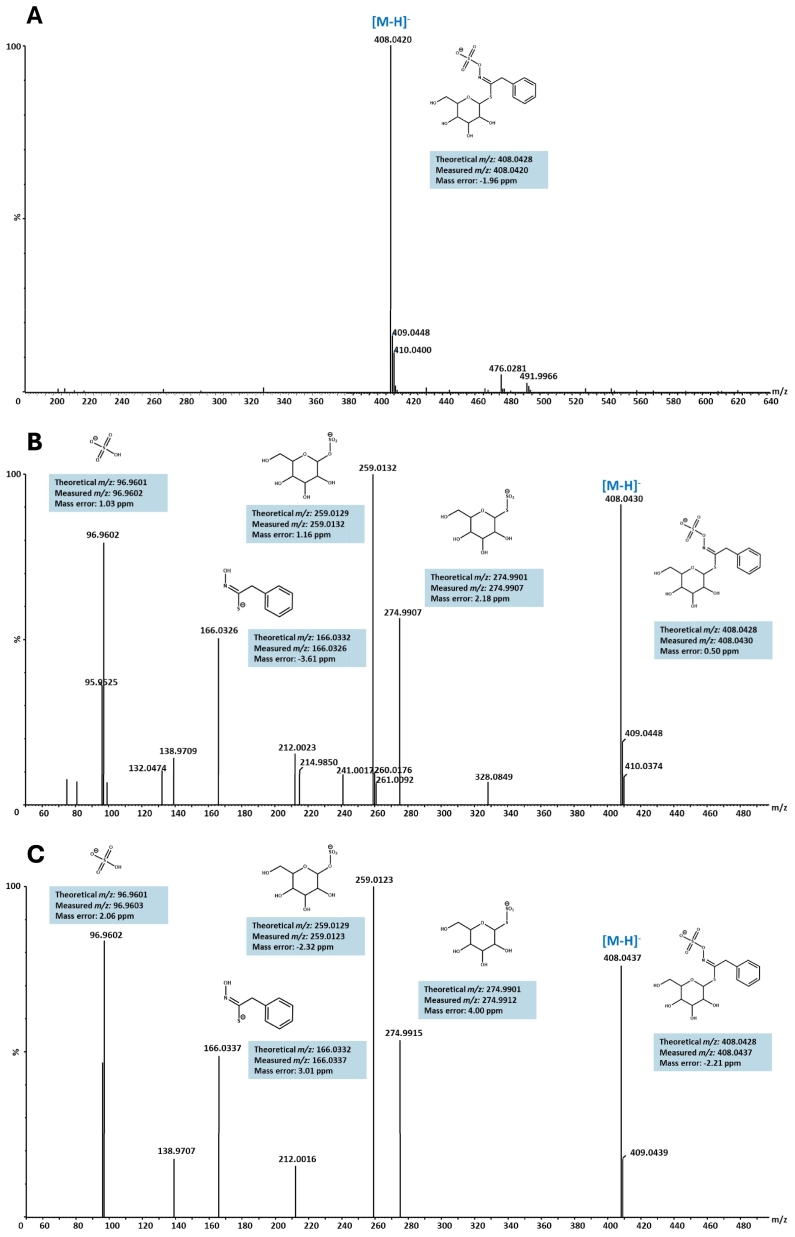
MS and MS/MS spectrum of glucotropaeolin. MS spectrum in Sample 7 (A), MS/MS spectrum in standard solution (B), and MS/MS spectrum in Sample 7 (C).

Initially, the GT and BITC quantification was performed using UHPLC-UV. In most samples, the levels of GT and related compounds were below the limit of quantification (LOQ), except for Sample 7 (Table 1, Fig. S3). Sample 7 contained 6.0 ± 0.4 μg/mL GT and was the only sample with quantifiable GT content by UHPLC-UV. Subsequent analyses were therefore carried out using UHPLC-HRMS, which provides limits of detection (LOD) and quantification (LOQ) that are approximately 100-fold lower than those of UHPLC-UV. The presence of GT in Sample 7 was confirmed by comparing it with an authentic standard via matching the retention time, the accurate mass of the precursor ion ([M- H]^−^ at *m/z* 408.0430), and the characteristic fragment ions (Bialecki et al., 2010), all with a mass accuracy below 5 ppm (Fig. 8).

A library containing over twenty biologically active substances likely to be found in ethanolic samples from different parts of the nasturtium plant was prepared based on the work of Česlová et al. (Česlová et al., 2023) and Garzón et al. (Garzón et al., 2015). Table 1 presents a list of individual food supplements, and the annotated phenolic compounds (Table S5) as determined by untargeted UHPLC-HRMS analysis in conjunction with the library. The analysis primarily revealed the presence of isomers of caffeoylquinic acid (1,2), along with isoquercitrin (4) and the derivatives of quercetin (5,6) and kaempferol (3), as isoquercitrin was the most abundant substance. The MS/MS spectra of the major phenolic compounds in the food supplements are presented in Fig. S4-S7.

### 3.5 Comparison of myrosinase activity

The effect of thermal processing on myrosinase activity is shown in Fig. S8. The highest rate of GT hydrolysis was observed in the temperature range of 40–60 °C. This indicates that temperatures up to 60 °C do not significantly denature the enzyme. Decreased activity was observed at 70 °C, and the enzyme was nearly completely inactivated at 80 °C. This, close to complete loss of activity at 80 °C, confirms the effectiveness of the boiling step used for deactivating the enzyme.

Next, we compared the activity of enzymes isolated from the pretreated seeds. The results are presented in Fig. S9. The enzyme isolated from the untreated sample exhibited the highest enzymatic activity, followed by those isolated from the freeze-dried and steam-treated samples. In contrast, the activity of the enzyme isolated from the boiled extract was almost negligible. These results align well with those obtained for the corresponding extracts, suggesting that the observed trends in myrosinase activity persist after enzyme isolation.

As shown in Fig. S10, myrosinase exhibited the highest activity in phosphate buffer at a pH of 6.0, followed by water. Increasing the concentration of ethanol resulted in a gradual decrease in myrosinase activity, with a complete loss of activity observed in 100% ethanol. An increase in activity within the plant matrix when the enzyme from Extract B was added to Extract BB suggested that nasturtium constituents may protect or stabilize solvent-induced denaturation and contribute to the maintenance or enhancement of myrosinase activity.

## 4 Discussion

This study investigated the stability and hydrolysis of GT in extracts from garden nasturtium seeds, leaves, and flower buds, to identify the factors that influence the final composition of GT degradation products in the extracts with an expected high GT and BITC content. Boiling halted GT hydrolysis, which is mostly accelerated by myrosinase, whereas steaming slowed hydrolysis compared to lyophilized and untreated extracts from the ground seeds. These findings are consistent with previous studies that have reported steaming to be an effective method of preserving GSs while reducing myrosinase activity. However, boiling can result in significant GS loss depending on the structure (Barba et al., 2016; Vieites-Outes et al., 2016). However, the effect of processing on myrosinase activity varies considerably among Brassicales species (Ghawi et al., 2012; Piekarska et al., 2013; Vancoillie et al., 2023). Steaming and microwave treatment reduced myrosinase activity in cabbage (Oloyede et al., 2021), but high-pressure treatment preserved enzyme activity in broccoli and mustard seeds under certain conditions (Okunade et al., 2020; Van Eylen et al., 2006). In contrast, myrosinase from green cabbage was reported to be highly susceptible to both thermal and high-pressure processing (Ghawi et al., 2012). Freeze-drying may reduce myrosinase activity, though it does not eliminate it completely (Doheny-Adams et al., 2017).

Myrosinase isolated from garden nasturtium exhibited thermal stability up to 50 °C. This stability exceeded that reported for myrosinase from broccoli juice and rapeseed sprouts, which remained stable only up to 40 °C (Van Eylen et al., 2007; Xie et al., 2022). However, it was lower than that observed for purified mustard seed myrosinase, which kept activity up to 60 °C (Van Eylen et al., 2006). Similar temperature-dependent increases and subsequent decreases in activity have also been reported for watercress and other Brassicales species (Aripin & Surugau, 2016; Okunade et al., 2020; Van Eylen et al., 2007). Exposure to higher temperatures generally results in irreversible denaturation and loss of activity (Van Eylen et al., 2006; Van Eylen et al., 2007).

A control sample pretreated with boiling water showed no GT degradation, which confirms complete thermal inactivation of the endogenous myrosinase. To verify whether GT degradation could be reinitiated, an active enzyme extract obtained from untreated seeds was added to the sample prepared in 70% ethanol. The low myrosinase activity observed in dried garden nasturtium seeds was consistent with the prolonged GT half-life measured in real samples (Extract B, t_1/2_ = 33 h). Furthermore, chopping and homogenisation were reported to reduce myrosinase activity through the mechanical and chemical damage of the enzyme (Piekarska et al., 2013). The lower activity observed in the present study compared to previous reports (Kleinwächter et al., 2008; Oloyede et al., 2021; Pardini et al., 2021; Restović et al., 2024) may be attributed to seed drying, storage prior to analysis, the absence of activating cofactors (e.g., ascorbic acid), and the use of 70% ethanol for extraction.

Importantly, the trends observed in the extracts are consistent with those obtained using the isolated myrosinase. This indicates that the two experimental approaches are in good agreement. Our results indicate rapid GT hydrolysis in the extracts (Fig. 1), with GT content in the seeds varying from 39.9 to 0.05 mg/g depending on the pretreatment method. Analysis of three different batches of untreated Extract B confirmed GT levels of 7.1–15.2 mg/g (RSD 31%), reflecting natural variation in GS content. The variability in secondary metabolite levels in garden nasturtium tissues is strongly influenced by climate and growing conditions (Česlová et al., 2023; Frei et al., 2024; Kleinwächter et al., 2008). Previous studies detailed the phenolic composition of nasturtium samples and the changes that occur during processing, including drying, and freezing, analysing GT in 200 individual plants. The dominant bioactive compound in all nasturtium tissues was GT. A decrease in GT content was observed in frozen samples compared to dried samples (Česlová et al., 2023). Since the samples were ground during processing, changes in GT content are likely to be related to the activation or deactivation of myrosinase.

As reported in the literature (Subramanian & Anandharamakrishnan, 2023), the ethanolic maceration of whole seeds increases the permeability of the cell membrane by disrupting its structure. This allows myrosinase to interact with GT and results in complete hydrolysis of GT within a few days. However, a potential drawback of long maceration periods is the decomposition of unstable compounds, including ITCs (Subramanian & Anandharamakrishnan, 2023). In our study, hydroethanolic treatment resulted in a myrosinase catalysed hydrolysis of GT, producing unstable BITC. Depending on the pH and temperature of the extraction, low levels of BC (Krell et al., 2022; Vrca et al., 2022) or benzyl thiocyanate can also form (Wielanek et al., 2004). However, neither BC nor BTC was detected in our samples. The formation of ITCs and nitriles is pH-dependent (Andernach et al., 2024). In *Tropaeolum majus* L. hair root cultures, a pH of 6.5–8 (neutral to slightly alkaline) was optimal for myrosinase activity towards BITC, while the conversion of GT to BC primarily occurred under acidic conditions (Wielanek et al., 2004). These findings support our observation that, at a neutral pH, GT was exclusively hydrolysed to BITC, with BC levels below the limit of detection (Table S3), when BC is formed via the thermal degradation of GT and is expected to be more thermally stable than BITC (Krell et al., 2022).

As previously mentioned, the unstable isothiocyanate functional group of BITC undergoes an addition reaction. In hydroethanolic solutions, BITC then undergoes a series of reactions that lead to the formation of benzyl thiocarbamic acid (DP1) and its dimerization product, 1,3-dibenzylthiourea (DP2) (Fig. S2). These findings are consistent with those of previous studies (Cirilli et al., 2020; Tian et al., 2016). In ethanol-containing systems, BITC may also form thiocarbamic acid esters (Cirilli et al., 2020). Water can act as a nucleophile, attacking the electrophilic carbon atom of the isothiocyanate group to form benzylamine. This can then react with BITC to produce 1,3-dibenzylthiourea (Pecháček et al., 1997; Tian et al., 2016). Similar degradation pathways were reported by other authors (De Nicola et al., 2013; Hanschen et al., 2014; Xu et al., 2021). The corresponding amine was identified as the primary degradation product at physiological pH (Andernach et al., 2024) and during heat treatment or drying in food matrices (Esparza et al., 2020). In our hydroethanolic extracts stored at laboratory temperature, benzylamine was found in small amounts, with its quantity remaining largely constant. Over time, it reacted with BITC to form 1,3-dibenzylthiourea (Pecháček et al., 1997; Tian et al., 2016).

Garden nasturtium extracts are commonly used by the public to treat urinary tract infections, for which BITC is a potentially active ingredient (Hanschen et al., 2014; Sofrata et al., 2011; Vrca et al., 2022). However, BITC was not detected in most of the commercially available extracts analysed in this study. Instead, the major constituents were phenolic compounds, including isoquercitrin and caffeoylquinic acid derivatives, indicating that the biological activity of these products is primarily attributable to these compounds. Consistently, Bazylko et al. (2013), reported that levels of BITC strongly depend on the way the extract is prepared, while the anti-inflammatory and antioxidant effects are largely attributable to phenolic compounds (Bazylko et al., 2013).

Drops, containing extracts from garden nasturtium, are widely used due to the documented bioactivity of BITC in the body (Vrca et al., 2022). It is important to inform consumers about the GT and BITC content of commercially available products, and about how to preserve GT and/or its hydrolysis products in both extracts and fresh preparations. The optimal GT/BITC ratio for human use remains an unresolved issue. It was suggested that a daily intake of at least 150 mg GT is required to achieve antibacterial BITC concentrations (Kleinwächter et al., 2008), which highlights the need for raw material with high GT content. GSs are converted to ITCs by plant myrosinase and the human gastrointestinal microflora. However, this process is generally inefficient and highly variable between individuals, with a higher conversion rate occurring during the day (Fahey et al., 2012). Furthermore, the bioavailability of BITC was reported to be higher compared to that of GT (Kühn et al., 2018). Although Extract BS had a similar BITC content profile similar to those of Extracts BL and B, GT hydrolysis occurred at a slower rate. Therefore, steam-pretreated seeds appear to provide the most favourable GT/BITC profile. In contrast, although extracts prepared from boiled seeds maintained high GT levels during storage, potential GT loss during the boiling step should also be considered. The maximum level of BITC in our extracts did not exceed 10 mg/g of seeds. As a daily intake of 400–570 mg BITC is considered safe (Dinh et al., 2021), the recommended doses of the extracts are unlikely to pose safety concerns. However, as GT degradation products such as 1,3-dibenzyl-2-thiourea and benzyl thiocarbamate may form in ethanolic extracts, the long-term health effects of these compounds require further investigation.

## 5 Conclusion and future perspective

This study demonstrates that GT in garden nasturtium can be preserved in edible extracts by controlling the activity of the enzyme myrosinase during processing. Without such control, GT rapidly hydrolyses to BITC, which is itself unstable and degrades further. BITC was found to be unstable over a period of nine weeks, degrading into benzyl thiocarbamate and 1,3-dibenzyl-2-thiourea, as confirmed by high-resolution mass spectrometry. Surprisingly, commercial extracts contained very low levels of GT and BITC, with phenolic compounds likely accounting for the majority of the observed biological effects.

Therefore, partial myrosinase inactivation is essential to maintain GT levels, for example, by controlled heating. These findings offer valuable insight for producing garden nasturtium-based food extracts, facilitating the development of functional foods and nutraceutical ingredients with consistent bioactivity profiles, stability, and efficacy.

## CRediT authorship contribution statement

**Lucie Chocholoušová Havlíková:** Writing – original draft, Resources, Methodology. **Marija Dmytryšyn:** Methodology, Investigation. **Hana Kočová Vlčková:** Methodology, Investigation, Data curation. **Štefan Kosturko:** Investigation. **Petr Chocholouš:** Writing – review & editing, Conceptualization. **František Švec:** Writing – review & editing, Supervision.

## Declaration of competing interest

The authors declare that they have no known competing financial interests or personal relationships that could have appeared to influence the work reported in this report.

## Data Availability

Experimental data are shared via Zenodo database under the reference  DOI: 10.5281/zenodo.20700242.
