# Supplementary material for: Processing-induced hydrolysis and degradation pathways of glucotropaeolin in edible garden nasturtium (Tropaeolum majus L.) extracts
Source: Food Chem X. 2026 Jul 1;37:104162. doi: 10.1016/j.fochx.2026.104162 (PMC13355715; doi:10.1016/j.fochx.2026.104162)
Supplement: Supplementary file 1 — Supplementary material [file mmc1.docx]

Supplementary information

Processing-induced hydrolysis and degradation pathways of glucotropaeolin in edible garden nasturtium (*Tropaeolum majus L.*) extracts

Lucie Chocholoušová Havlíková, Mariya Dmytryshyn, Hana Kočová Vlčková, Štefan Kosturko, Petr Chocholouš, František Švec

Charles University, Faculty of Pharmacy in Hradec Králové, Department of Analytical Chemistry, Heyrovského 1203, 500 03 Hradec Králové, Czech Republic

[chocholousova@faf.cuni.cz](mailto:chocholousova@faf.cuni.cz)

Corresponding author:

Lucie Chocholoušová Havlíková

Department of Analytical Chemistry

Faculty of Pharmacy, Charles University

Heyrovského 1203

Hradec Králové 500 03

Czech Republic

Tel.: +420495067354; fax: +420495518718

E-mail address: [chocholousova@faf.cuni.cz](mailto:chocholousova@faf.cuni.cz)

**S1. Materials and methods**

**S1.1. Sample preparation**

Table S1:

Sample preparation.

| **Garden nasturtium -Extract** | | **Step 1- Pretreatment** | | **Step 2 - Extraction** | **Step 3 - Time of sampling** | **Step 4 - Chromatography** | | |
| --- | --- | --- | --- | --- | --- | --- | --- | --- |
| **Seeds** | **Extract A** | | | 15.0 g + 100.0 mL 50% EtOH | 0,1,2,3,4,8,12,16 week | 10 x dilution  70% EtOH | Filtration 0.22 µm | UHPLC-UV analysis |
|  | **Extract BB** | 50.0 g + 100.0 mL **boiling** water 5 min, decanted, air-dried | grinding | 0.5 g + 50.0 mL 70% EtOH, **sonication** 10 min | 0, 4, 24, 48, 72, 96 h,  1, 2, 3, 4 week |  |  |  |
|  | **Extract BS** | 50.0 g **steam** (70 °C) 15 min, air-dried |  |  |  |  |  |  |
|  | **Extract BL** | **frozen** at -29 °C, **sublimated** at -55 °C under vacuum 24 h |  |  |  |  |  |  |
|  | **Extract B** |  |  |  |  |  |  |  |
| **Leaves** | | 10 pc homogenised | chopped 0.5 cm pc | 0.1 g +10.0 mL 70% EtOH, **sonication** 10 min | 0, 4, 24, 48, 72, 96, 144 h |  |  |  |
| **Flower buds** | |  |  |  |  |  |  |  |
| **Food supplements** | | | | | 0 h |  |  |  |
| w: weeks, h: hours, EtOH: ethanol, pc: pieces | | | | | | | | |

**S1.2. Ultra-high performance liquid chromatography UV detection**

Analyses were carried out using the Nexera X2 UHPLC system (Shimadzu Corporation, Japan). This system included a CBM-20A communication module, two LC-30AD solvent delivery systems, a DGU-20 A5R degassing unit, a SIL-30AS autosampler, a CTO-20AC column oven, and a SPD-M30A diode array detector. LabSolution software (Shimadzu Corporation, Japan) was used to collect and evaluate the data. The stationary phase was Ascentis Express Phenyl-Hexyl 100 x 4.6 mm, 5 µm column (Supelco, Czech Republic). The mobile phase consisted of acetonitrile (component A) and 0.085% aqueous phosphoric acid solution (component B), which was pumped at a flow rate of 1 mL/min. The gradient profile started at 5% A and increased to 25% (A) over 5 min. The percentage of acetonitrile in the mobile phase increased more steeply to 100% over the next 5 min. Subsequently, the percentage of acetonitrile returned to the initial conditions. The system was then equilibrated for 3 min. Spectrophotometric detection in UV was set at 220 nm for data acquisition. A 3 µL sample was injected into the UHPLC system.

**S1.3. Ultra-high performance liquid chromatography – high-resolution mass spectrometry**

The food supplements and garden nasturtium extract A (more details in section 2.2. in the manuscript) were analysed using the UPLC I-Class system (Waters, USA) coupled with the high-resolution mass spectrometer Synapt G2-Si quadrupole time-of-flight (HRMS) (Waters, USA). A 2 µL sample was injected into an Acquity BEH C18 separation column (2.1 × 50 mm; 1.7 µm, Waters, USA). The mobile phase flow rate was set at 0.40 mL/min. Gradient elution was performed using a mobile phase consisting of 0.1% aqueous formic acid as component A and acetonitrile as component B. The gradient profile started with a linear increase from 5 to 25% B over 2.5 min, followed by a ramp from 25 to 95% B in another 2.5 min, and ended with a re-equilibration step in 5% B for 2 min. The total chromatographic run time was 7 min. The electrospray ionization (ESI) source was operated in both negative and positive modes, with the following parameters: a capillary voltage of -1.0 kV/2.5kV, a sampling cone voltage of 10 V, a source offset of 20 V, and a source temperature of 130 °C. The nitrogen desolvation stream was set to a flow rate of 1000 L/h at a temperature of 600°C. Nitrogen was also used as a cone gas at a flow rate of 50 L/h, and argon was used as a collision gas. The nebulization gas pressure was maintained at 6.5 bar.

An additional targeted MS/MS scan of the deprotonated GT in Extract A (more details in section 2.2. in the manuscript) was additionally acquired at a collision energy of 125 eV. Using the data-independent acquisition approach, which collects MS and MS/MS data concurrently, other substances and benzyl isothiocyanate degradation intermediates were detected. MS spectra were acquired over the m/z range of 50–1200 with a fixed collision energy of 4 eV for both polarity MS scans, and a ramped collision energy ranging from 10 to 40 eV for both MS/MS polarity scans. Leucine enkephalin (200 pg/µL) was used as an internal calibrant, and a 0.5 mmol/L aqueous sodium formate solution was used as an external calibrant. MS data acquisition and processing were conducted using MassLynx 4.1 software (Waters, USA), and analyte identification and annotation were conducted using the UNIFI Scientific Information System Software (Waters, USA). Two main annotation criteria were employed (i) precursor ion mass accuracy below 5 ppm and (ii) identification of characteristic fragment ions with mass accuracy also below 5 ppm. Retention time was used as the third criterion for identification.

**S1.4. UHPLC-UV Method Validation**

The applicability of the developed UHPLC method was verified by the system suitability test (SST) and method validation. The SST used the working standard solutions containing 50 µg/mL of GT, 25 µg/mL of BC, 12.5 µg/mL of BTC, and 25 µg/mL of BITC. The repeatability of retention times and peak areas (expressed as relative standard deviation), peak symmetry, and peak resolution were evaluated according to European Pharmacopoeia (European Pharmacopoeia, 2022). Method validation included linearity, precision, and accuracy using garden nasturtium seed Extract A, at an extraction time of 24 h. The standard addition method at three concentration levels was used to evaluate method accuracy, expressed as a percentage recovery, as well as the method precision. The samples were spiked with GT, BC, BTC, and BITC at three concentration levels within the following ranges: 10-50 µg/mL for GT and 5-10 µg/mL for BC, BTC, and BITC, respectively. Method precision included evaluating repeatability of the method and its inter-day precision. Repeatability was calculated as % RSD for three replicates that were individually prepared at three different concentration levels. Inter-day precision was evaluated on three consecutive days, with three replicates at one concentration level. Linearity was calculated using the least squares method (ICH, 2005) and eight calibration points within the following ranges: 2-200 µg/mL for GT, 2.5-60 µg/mL for BC, 0.5-15 µg/mL for BTC, and 1-150 µg/mL for BITC. Matrix linearity was evaluated using garden nasturtium seeds spiked at the same concentrations.

**S2. Results**

**S2.1. UHPLC-UV method development**

During method development, separation columns with C18 and phenyl hexyl stationary phases were tested. The key factors were the complete resolution of glucotropaeolin (GT) from the other compounds in the extracts and the GT peak shape. Acetic, formic, and phosphoric acids were tested to adjust the pH of the aqueous part of the mobile phase. When acetic and formic acids were used, GT eluted as a double peak. Using only phosphoric acid and the phenyl-hexyl stationary phase resulted in a satisfactory GT peak shape. However, the GT peak shape was also strongly affected by the type of sample solvent. The initial mobile phase contained more than 90% of the aqueous part of the mobile phase because GT is polar and its hydrolysis products are more lipophilic. The acetonitrile gradient first increased slowly to separate GT from other unidentified compounds in the extracts and then increased more rapidly to a higher acetonitrile content to elute the hydrolysis products. The total analysis time, including the equilibration of the analytical column, was 12 min. During the method development, we also tested HILIC mode using an Ascentis Express OH5 analytical column (100 x 3.0 mm, 2.7 µm). The GT was eluted and separated from the other compounds. However, the hydrolysis products eluted at the system void volume. For this reason, the HILIC mode was omitted. Considering the UV spectra of the monitored compounds, the detection wavelength was set to 220 nm.

The UHPLC-UV analysis of the working standard solution is presented in Fig. S1. Working solutions were prepared at the following concentrations: 50 µg/mL for GT, 25 µg/mL for benzyl cyanide (BC), 12.5 µg/mL for benzyl thiocyanate (BTC), 25 µg/mL for benzyl isothiocyanate (BITC), and 10 µg/mL for 1,3-dibenzyl-2-thiourea.


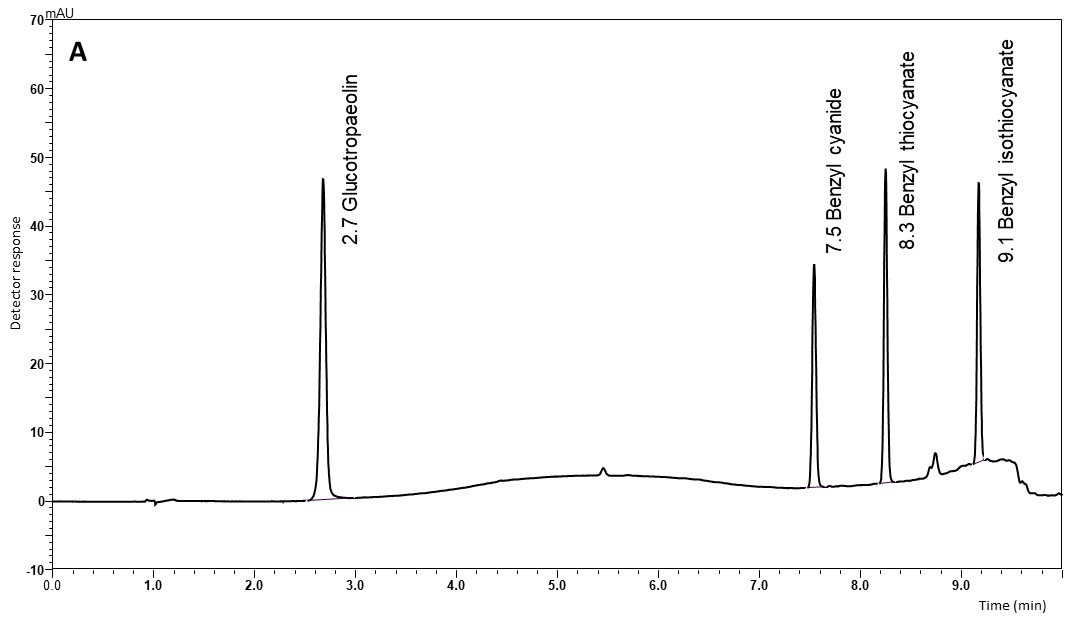


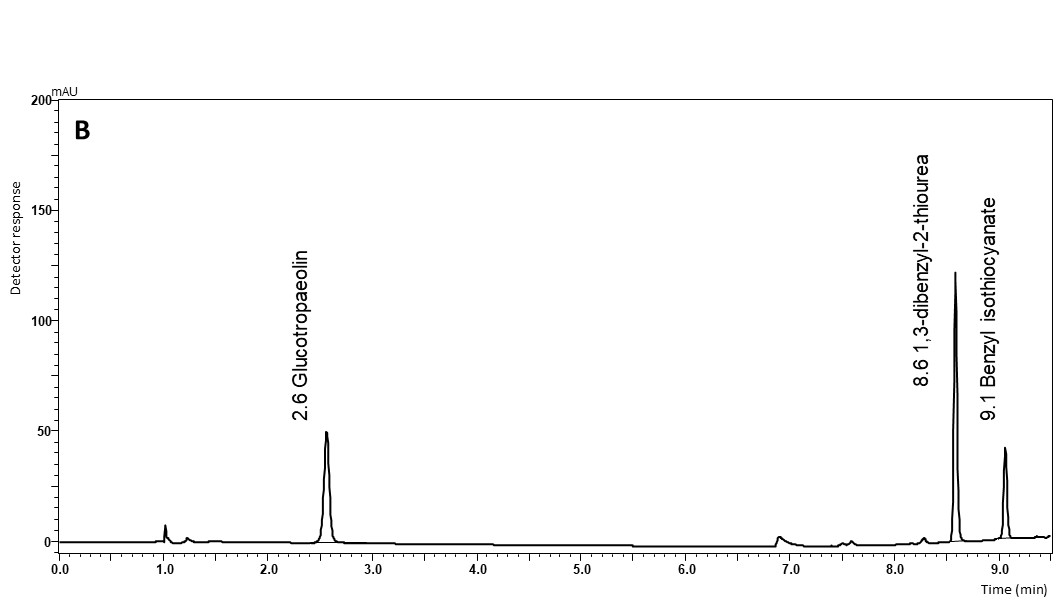


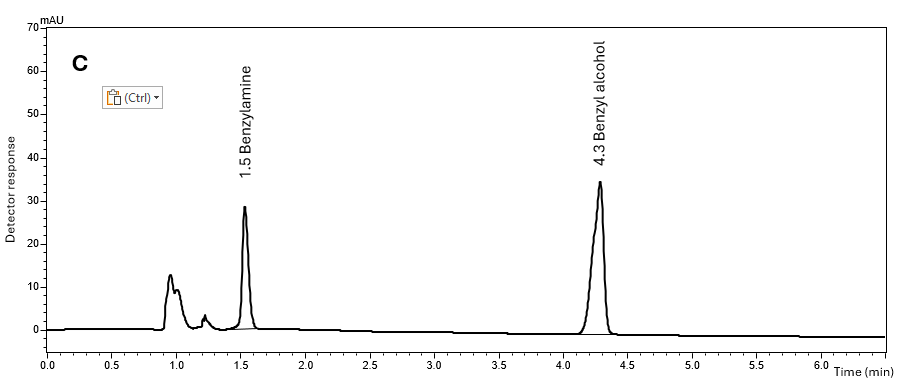


Figure S1:

Chromatogram of UHPLC-UV analysis of standard solution. (A) GT (50 µg/mL) and its possible products of hydrolysis – benzyl isothiocyanate (25 µg/mL), benzyl cyanide (25 µg/mL), and benzyl thiocyanate (12.5 µg/mL). (B) GT; benzyl isothiocyanate - product of hydrolysis of glucotropaeolin in Garden nasturtium; 1,3-dibenzyl-2-thiourea (10 µg/mL) - degradation intermediate of benzyl isothiocyanate. (C) Benzylamine (50 µg/mL) and benzyl alcohol (25 µg/mL) - products of hydrolysis of benzyl isothiocyanate in Garden nasturtium.

**S2.2. Method validation results**

For the SST, a working standard solution containing 50 µg/mL of GT, 25 µg/mL of BC, 12.5 µg/mL of BTC, and 25 µg/mL of BITC, was injected six times. The calculated relative standard deviations (% RSD) of the peak areas were less than 0.2 %, and the RSDs of the retention times were less than 0.1%. Peak resolution was in the range of 11-58, and the peak symmetry was 1.0 for all the analytes (see Table S2). Eight calibration points covered the tested range chosen for the linearity evaluation. A regression line was calculated using the method of least squares for the standard and matrix calibration ranges. The matrix regression line was obtained using spiked garden nasturtium seeds. A coefficient of determination greater than 0.998 for both lines confirmed the linear range. The detection and quantification limits were determined using a signal-to-noise approach. The signal-to-noise ratio was determined at expected location of the peak of interest. Table S3 presents the linearity range, coefficients of determination, y-intercept, slope of the regression line, and LODs and LOQs of the proposed method. The precision of the method was determined using individually prepared samples of garden nasturtium seeds that were spiked individually with the stock standard solutions to reach the final concentration levels of 10, 25, and 50 µg/mL for GT and BITC, and 5, 7.5, and 10 µg/mL for BC and BTC, respectively. Each sample was prepared in triplicate. Intraday precision, carried out over three consecutive days, at a concentration of 10 µg/mL for each analyte in triplicate, was lower than 5.0 %. The standard addition method was also used to evaluate the method accuracy at the same concentration levels. The recovery of the method was in the range of 95.2-104.1%. The method accuracy, precision, and interday precision are summarized in Table S2.

Table S2:

System suitability test including retention time, area, peak resolution, symmetry factor, and peak capacity, as well as method precision and accuracy achieved under the optimized conditions.

|  | Analyte | | |  |
| --- | --- | --- | --- | --- |
| Parameter | GT | BITC | BC | BTC |
| SST^a^ |  |  |  |  |
| Retention time | 2.7 | 9.2 | 7.5 | 8.2 |
| Repeatability – t_R_ | 0.1 | 0.1 | 0.1 | 0.1 |
| Repeatability – areas | 0.1 | 0.1 | 0.2 | 0.2 |
| Peak capacity | 76 | 117 | 138 | 133 |
| Peak resolution | 24 | 15 | 58 | 11 |
| Symmetry factor | 1.0 | 1.0 | 1.0 | 1.0 |
| Validation |  |  |  |  |
| Accuracy^b^ Recovery (%) |  |  |  |  |
| Level 1 | 101.1 | 98.7 | 98.0 | 95.2 |
| Level 2 | 100.2 | 97.4 | 99.3 | 102.3 |
| Level 3 | 96.5 | 98.7 | 104.1 | 100.3 |
| Precision^b^ (RSD %) |  |  |  |  |
| Level 1 | 10.3 | 8.1 | 2.8 | 3.2 |
| Level 2 | 10.5 | 3.0 | 3.3 | 1.1 |
| Level 3 | 8.1 | 4.1 | 0.4 | 1.3 |
| Inter-day precision^c^ (RSD %) | 4.2 | 3.8 | 4.6 | 3.7 |
| ^a^ Six injections, GT 50 µg/mL, BC 25 µg/mL, BTC 12.5 µg/mL, and BITC 25 µg/mL | | | | |
| ^b^ Three samples injected three times each, GT and BITC: Lev1 10 µg/mL, Lev2 25 µg/mL, Lev 3 50 µg/mL, BC and BTC: Lev1 5 µg/mL, Lev2 7.5 µg/mL, Lev 3 10 µg/mL  ^c^ Three consecutive days, three replicates, 10 µg/mL | | | | |

Table S3:

Method linearity – standard and matrix calibration plots.

| Analyte | Linearity - Standard calibration | | | | | | Linearity - Matrix calibration | | | |
| --- | --- | --- | --- | --- | --- | --- | --- | --- | --- | --- |
|  | Range  [µg/mL] | R^2^ | Intercept | Slope | LOD  [µg/mL] | LOQ  [µg/mL] | Range  [µg/mL] | R^2^ | Intercept | Slope |
| GT | 2-200 | 0.999 | -5554 ±2105 | 3690 ±21 | 1.0 | 0.2 | 2-200 | 0.999 | -1158 ±2701 | 4032 ±48 |
| BC | 2.5-60 | 0.999 | 1818 ±717 | 16457 ±113 | 0.3 | 0.1 | 2.5-60 | 0.999 | 3476 ±2080 | 16807 ±270 |
| BTC | 0.5-15 | 0.999 | 411 ±555 | 8927 ±78 | 0.3 | 0.1 | 0.5-15 | 0.999 | 144 ±1163 | 8270 ±159 |
| BITC | 1-150 | 0.998 | 5489 ±2632 | 3231 ±50 | 0.3 | 0.1 | 1-150 | 0.998 | 3805 ±2166 | 3537 ±43 |

**S2.3. Stability of GT and BITC**

The stability of GT and BITC was monitored over a 25-day period in a 70% ethanol solution at room temperature and at concentration levels of 25, 50, and 100 µg/mL. The results summarized in Table S4 demonstrate that GT remained stable throughout the investigated period, with measured concentrations varying by less than ±5% from the initial concentration. In contrast, BITC gradually degraded over time, forming two degradation products, DP1 and DP2, whose retention times corresponded to those observed in the real samples.

Table S4:

|  | GT | | | BITC | | |
| --- | --- | --- | --- | --- | --- | --- |
|  | 25 µg/mL | 50 µg/mL | 100 µg/mL | 25 µg/mL | 50 µg/mL | 100 µg/mL |
| Time 0 | 100.0 | 100.0 | 100.0 | 100.0 | 100.0 | 100.0 |
| 5 days | 100.3±2.6 | 101.6±1.6 | 103.4±1.5 | 96.5±3.2 | 96.1±3.8 | 98.1±2.1 |
| 11 days | 98.8±3.1 | 101.6±2.1 | 99.9±2.8 | 86.9±3.6 | 87.7±4.2 | 92.6±2.5 |
| 18 days | 103.0±2.7 | 104.3±0.6 | 104.0±0.7 | 70.3±4.0 | 74.2±4.8 | 82.5±3.7 |
| 25 days | 103.3±2.6 | 103.9±1.0 | 103.8±1.2 | 59.2±4.5 | 63.9±4.6 | 73.3±4.4 |

Stability of glucotropaeolin and benzyl isothiocyanate in 70% ethanol during storage at room temperature, expressed as a percentage of the initial concentration. n=3

**S2.4. Hydrolysis of glucotropaeolin in garden nasturtium samples**

Glucotropaeolin (GT), underwent enzymatic cleavage to produce D-glucose and an unstable (Z)-thiohydroximate-O-sulfonate (Blažević et al., 2020). The resulting aglycone decomposed spontaneously at neutral pH to isothiocyanate (Hanschen et al., 2014). Benzyl isothiocyanate is an unstable compound in 50% and in 70% ethanol it undergoes hydrolysis to form two main degradation intermediates benzyl thiocarbamate and 1,3-dibenzyl-2-thiourea (Fig. S2A and S2B). The degradation pathway is similar to the proposed degradation pathway of sulforaphane (Tian et al., 2016).


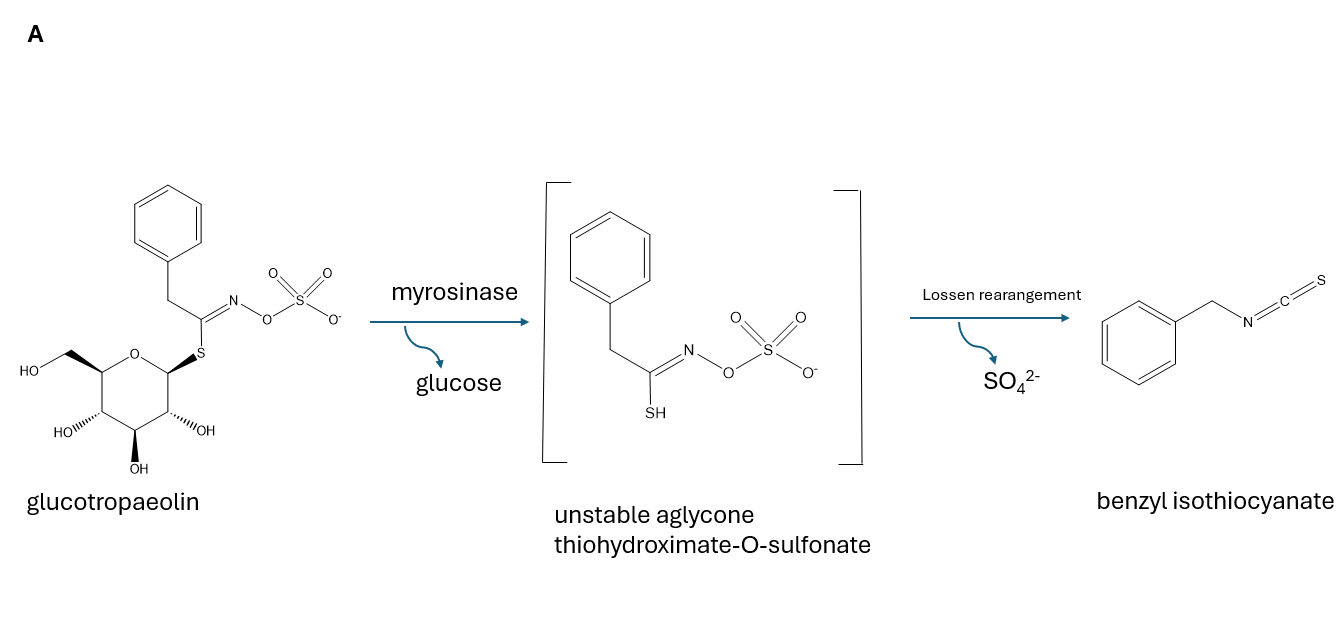


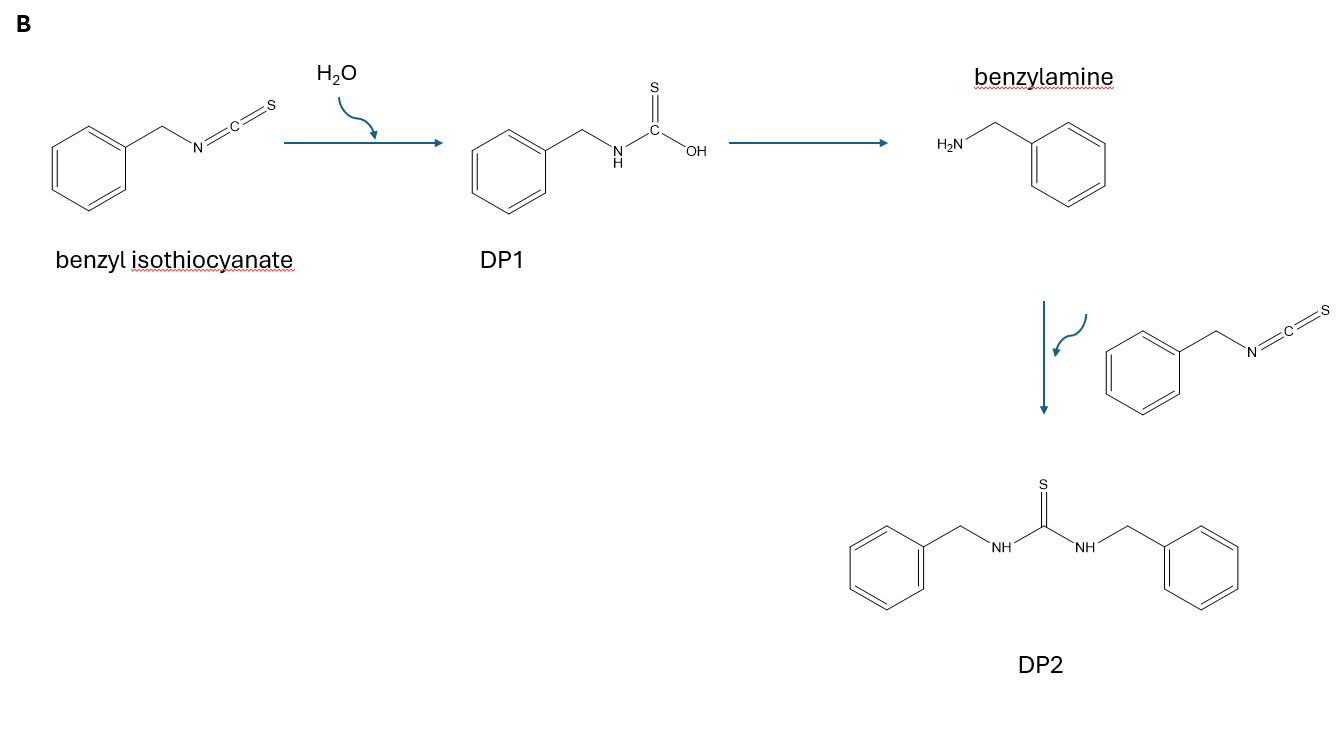


Figure S2:

Proposed enzymatic hydrolysis of glucotropaeolin in garden nasturtium (A) and proposed simplified mechanism of the degradation of benzyl isothiocyanate (B).

**S2.5. Profile of compounds in food supplements**


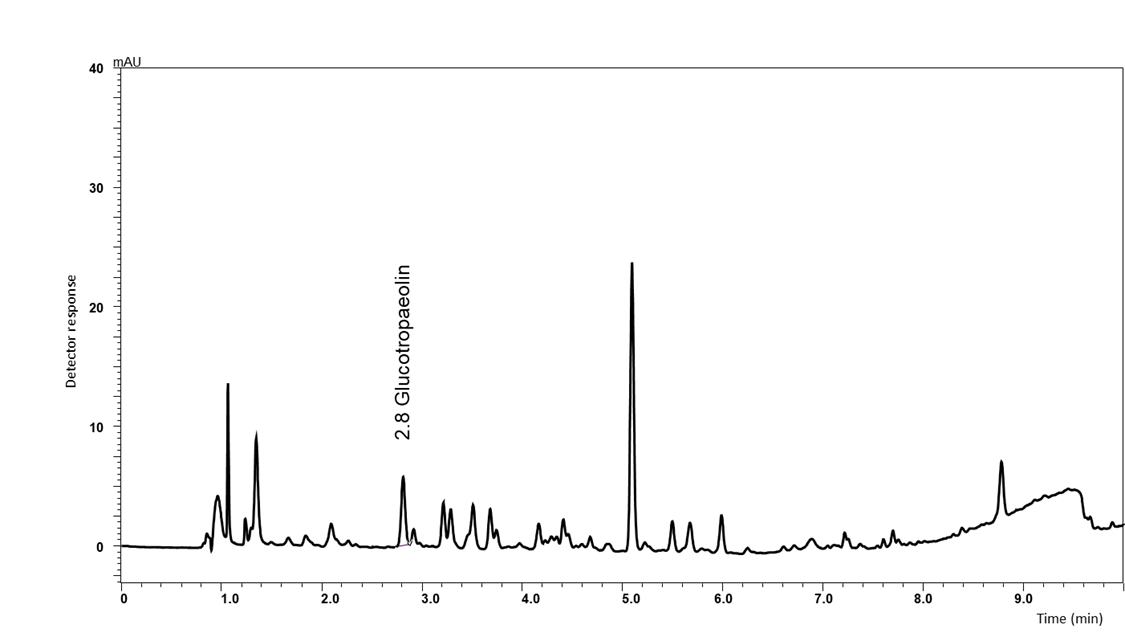


Figure S3:

UHPLC-UV analysis of Garden nasturtium commercial herbal extract (Sample 7). Conditions: Ascentis Express Phenyl-Hexyl column 100 x 4.6 mm, 5 µm, using a gradient of 0.085% aqueous phosphoric acid and acetonitrile (more details in section S1.4.), column oven at 30°C, and UV detection at 220 nm.

Table S5:

Summary of compounds identified in Samples 1–7, including retention times, deprotonated molecule ([M–H]⁻), fragment ions observed in HRMS, and corresponding figure numbers.

| Retention time (min) | Compound | [M-H]^-^ | Fragment ions | Figure |
| --- | --- | --- | --- | --- |
| 0.97 | Glucotropaeolin | 408.0420 | 274.9907, 259.0132, 166.0326 | 8 |
| 0.97 | Caffeoylquinic acid isomer 1 | 353.0878 | 191.0566, 179.0346, 135.0446 | S4 |
| 1.30 | Caffeoylquinic acid isomer 2 | 353.0878 | 191.0566, 179.0346, 135.0446 | S4 |
| 1.98 | Kaempferol dihexoside | 609.1447 | 429.0796, 285.0391, 284.0317 | S5 |
| 2.17 | Isoquercitrin | 463.0875 | 301.0340, 300.0273, 271.0244, 255.0292, 151.0032 | S7 |
| 2.23 | Quercetin deoxyhexoside 1 | 447.0927 | 301.0341, 300.0276, 271.0241, 255.0287, 151.0030 | S6 |
| 2.47 | Quercetin deoxyhexoside 2 | 447.0927 | 301.0341, 300.0276, 271.0241, 255.0287, 151.0030 | S6 |


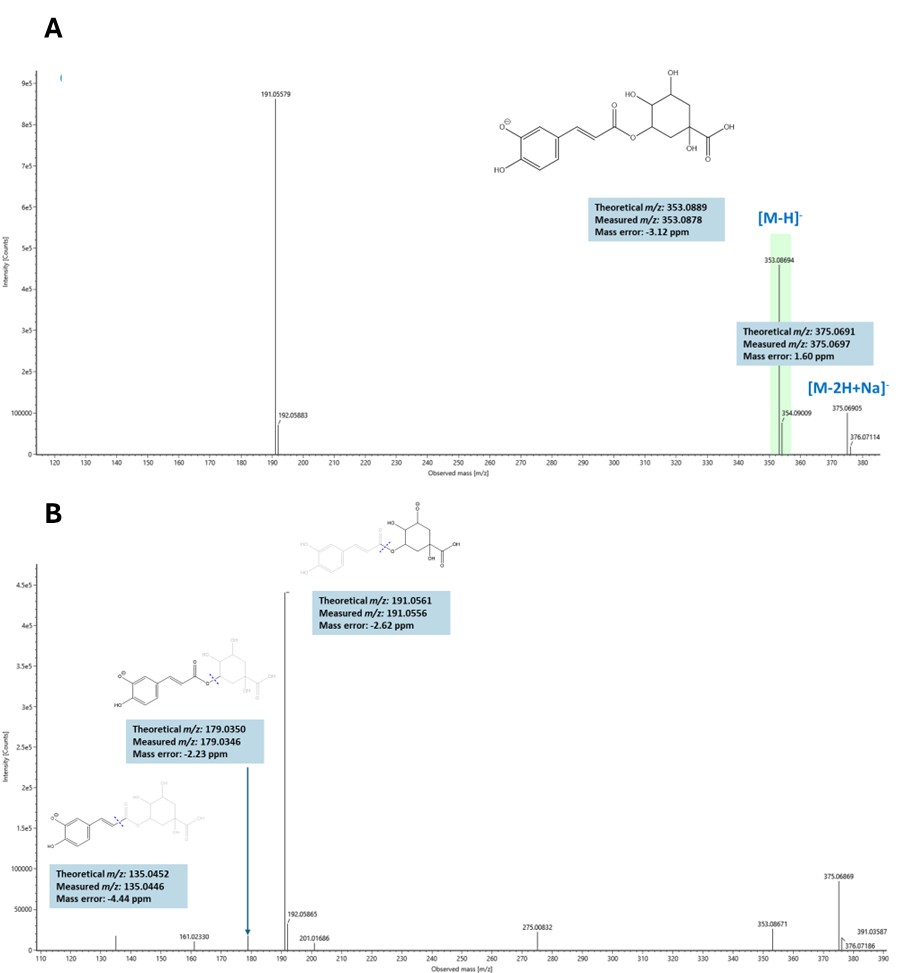


Figure S4:

Low energy MS spectrum (A) and high energy MS spectrum of caffeoylquinic acid isomer (B).


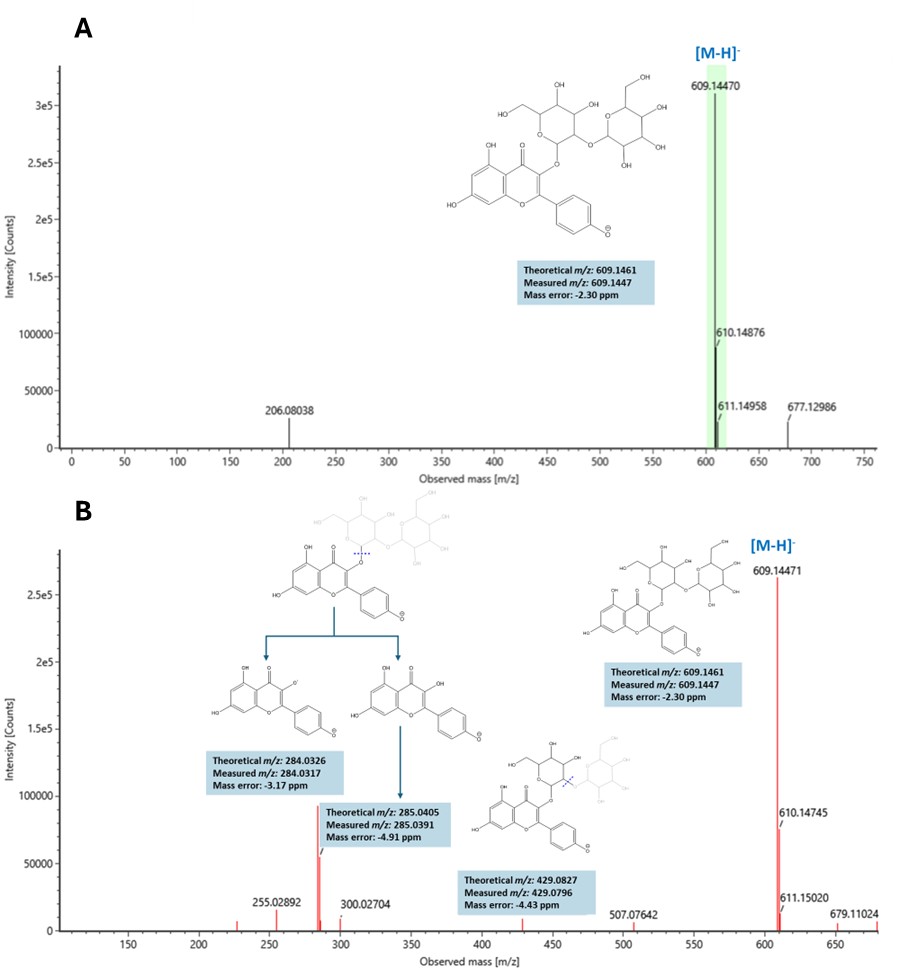


Figure S5:

Low energy MS spectrum (A) and high energy MS spectrum of kaempferol dihexoside (B).


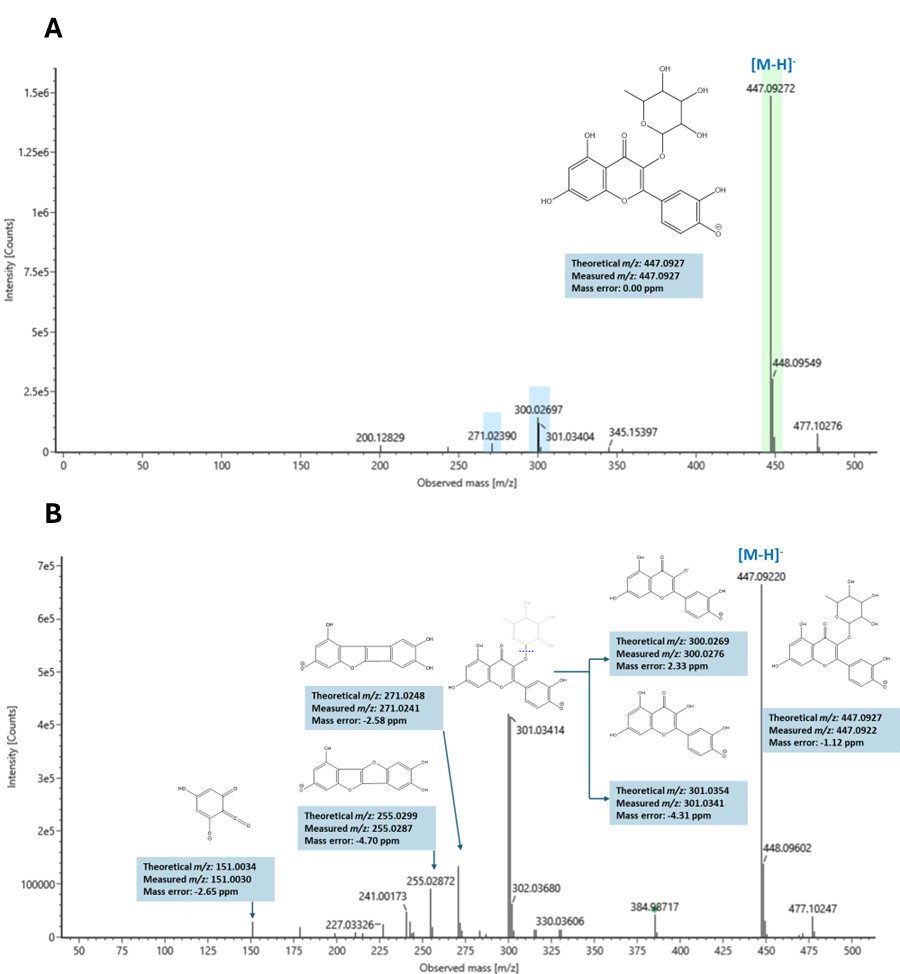


Figure S6:

Low energy MS spectrum (A) and high energy MS spectrum of quercetin deoxyhexoside (B).


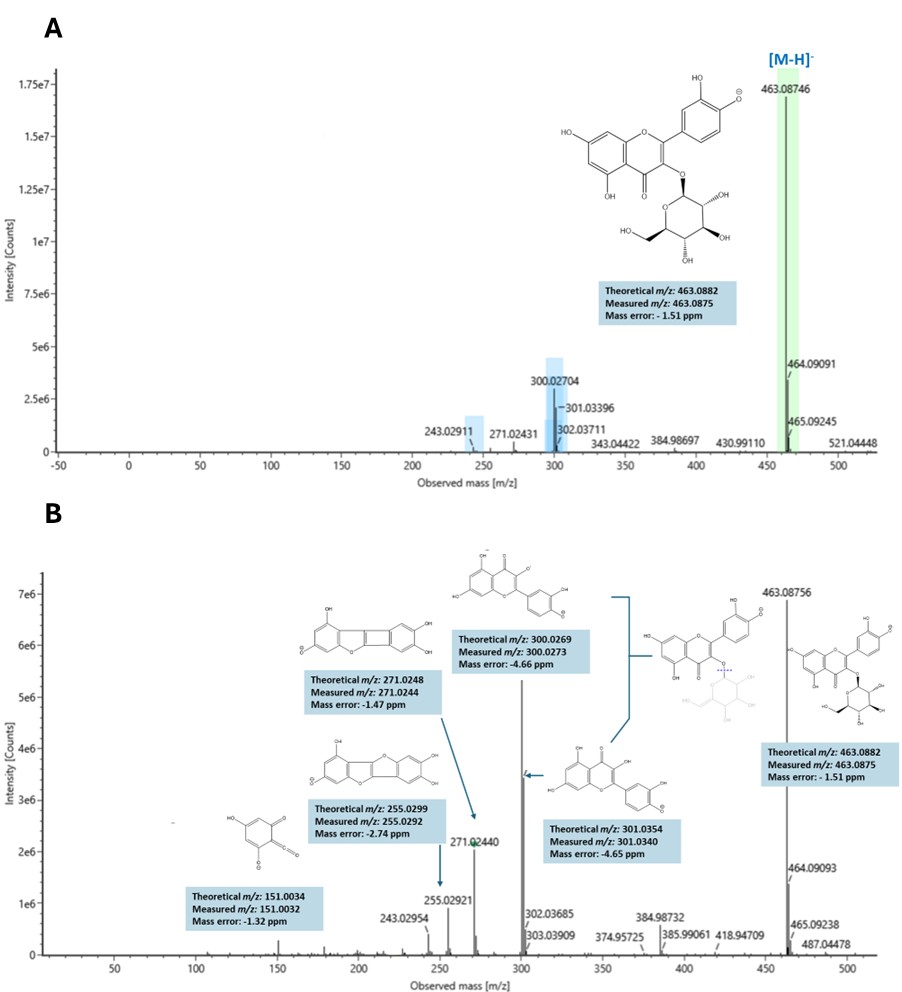


Figure S7:

Low energy MS spectrum (A) high energy MS spectrum of isoquercitrin (B).

S2.6. Comparison of Myrosinase activity after thermal treatment


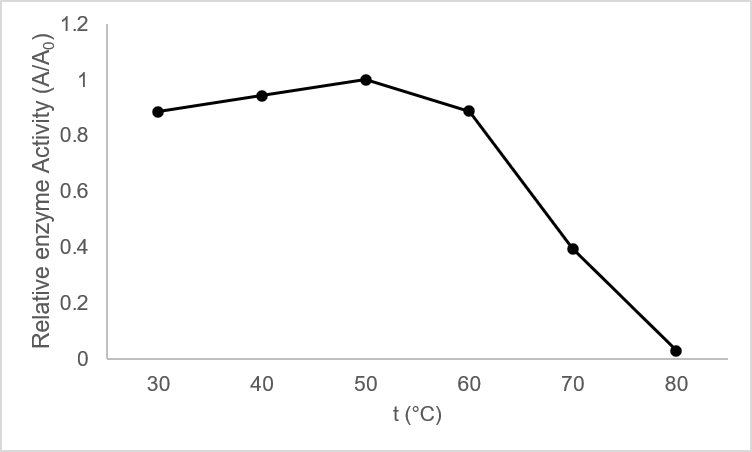


Figure S8:

Comparison of relative myrosinase activity after thermal treatment in 25 mM phosphate buffer (pH 6.0), A_o_ - highest enzyme activity at 50°C, A – myrosinase activity.


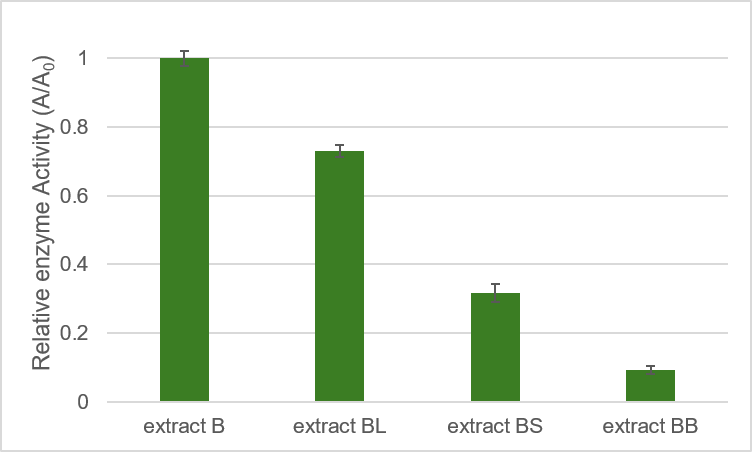


Figure S9:

Comparison of relative myrosinase activity of myrosinase isolated from pretreated seeds by boiling, steaming, lyophilization, and from untreated seeds, A_o_ - highest myrosinase activity from untreated seeds in 25 mM phosphate buffer (pH 6.0), A - myrosinase activity


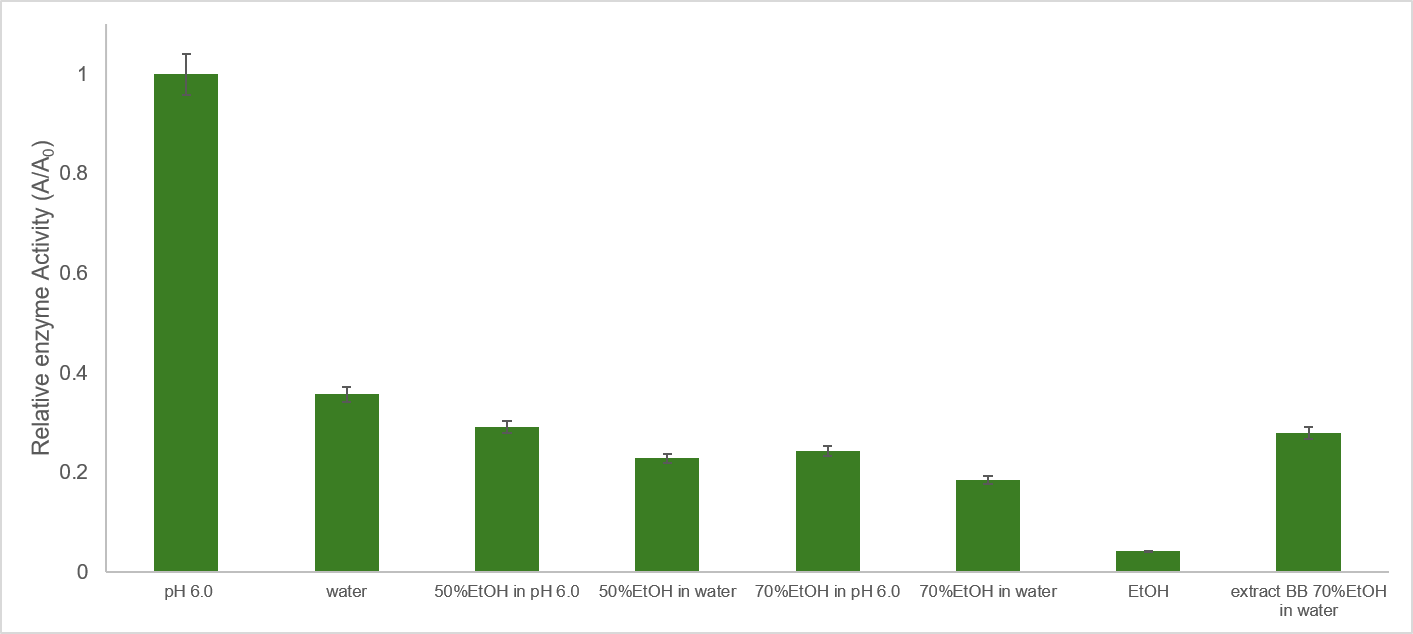


Figure S10:

Comparison of relative myrosinase activity - influence of the extraction medium on myrosinase activity (A), A_o_ - highest enzyme activity in pH 6.0, pH6.0: 25 mM phosphate buffer (pH 6.0).

**References**

Blažević, I., Montaut, S., Burčul, F., Olsen, C. E., Burow, M., Rollin, P., & Agerbirk, N. (2020). Glucosinolate structural diversity, identification, chemical synthesis and metabolism in plants. *Phytochemistry*, *169*, 112100. <https://doi.org/https://doi.org/10.1016/j.phytochem.2019.112100>

European Pharmacopoeia, E., 11th ed. (2022). *chapter 2.9.5, 2.2.46, accessed 20 July 2025*.

Hanschen, F. S., Lamy, E., Schreiner, M., & Rohn, S. (2014). Reactivity and stability of glucosinolates and their breakdown products in foods. *Angew Chem Int Ed Engl*, *53*(43), 11430–11450. <https://doi.org/10.1002/anie.201402639>

ICH. (2005). International conference on harmonization, Geneva, Q2 (R1): Validation of analytical procedures: text and methodology. 1–13.

Tian, G., Li, Y., Cheng, L., Yuan, Q., Tang, P., Kuang, P., & Hu, J. (2016). The mechanism of sulforaphene degradation to different water contents. *Food Chemistry*, *194*, 1022–1027. <https://doi.org/https://doi.org/10.1016/j.foodchem.2015.08.107>
